# Increased airway glucose increases airway bacterial load in hyperglycaemia

**DOI:** 10.1038/srep27636

**Published:** 2016-06-08

**Authors:** Simren K. Gill, Kailyn Hui, Hugo Farne, James P. Garnett, Deborah L. Baines, Luke S.P. Moore, Alison H. Holmes, Alain Filloux, John S. Tregoning

**Affiliations:** 1 Mucosal Infection & Immunity Group, Section of Virology, Imperial College London, St Mary’s Campus, London, W2 1PG, UK; 2 MRC Centre for Molecular Bacteriology and Infection, Department of Life Sciences, Imperial College London, London, SW7 2AZ, UK; 3 Airway Disease Infection Section, National Heart & Lung Institute, Imperial College London, London, W2 1PG, UK; 4 Institute for Infection and Immunity, St George’s, University of London, London SW17 0RE, UK; 5 Health Protection Research Unit in Healthcare Associated Infection and Antimicrobial Resistance, Imperial College London, Hammersmith Campus, London W12 0HS, UK

## Abstract

Diabetes is associated with increased frequency of hospitalization due to bacterial lung infection. We hypothesize that increased airway glucose caused by hyperglycaemia leads to increased bacterial loads. In critical care patients, we observed that respiratory tract bacterial colonisation is significantly more likely when blood glucose is high. We engineered mutants in genes affecting glucose uptake and metabolism (*oprB*, *gltK*, *gtrS* and *glk*) in *Pseudomonas aeruginosa*, strain PAO1. These mutants displayed attenuated growth in minimal medium supplemented with glucose as the sole carbon source. The effect of glucose on growth *in vivo* was tested using streptozocin-induced, hyperglycaemic mice, which have significantly greater airway glucose. Bacterial burden in hyperglycaemic animals was greater than control animals when infected with wild type but not mutant PAO1. Metformin pre-treatment of hyperglycaemic animals reduced both airway glucose and bacterial load. These data support airway glucose as a critical determinant of increased bacterial load during diabetes.

Diabetes is associated with an increased risk of bacterial lung infection, especially when there are underlying chronic lung conditions^1^,^2^. It has recently been proposed that uncontrolled hyperglycaemia in diabetics increases lung glucose, providing a richer growth medium that facilitates bacterial infection^3^. Of note, diabetes where glycaemia is controlled is not associated with increased risk of bacterial infection^4^. Levels of glucose in the lungs are tightly regulated and are up to 12 times lower in the airway surface liquid lining the lungs than circulating levels^5^. Glucose concentration in the airway is kept low by the action of facilitative glucose transporters GLUT -1, 2 & GLUT-10^6^,^7^, highlighting the importance of limiting glucose availability in the lungs.

In differentiated airway cell culture models, it has been observed that increasing the basolateral glucose concentration increases the growth of the respiratory pathogens *Staphylococcus aureus*^8^ and *Pseudomonas aeruginosa*^6^,^8^,^9^. The aim of the current study was to determine the effect of glucose on bacterial growth *in vivo* and therefore directly link bacterial glucose metabolism with increased respiratory tract bacterial load in hyperglycaemia. To investigate this we used *P. aeruginosa*, a Gram-negative opportunistic bacterial pathogen, which is a major cause of respiratory infections. Chronic *P. aeruginosa* infections are most commonly associated with patients with cystic fibrosis (CF) and chronic obstructive pulmonary diseases (COPD)^10^, but importantly *P. aeruginosa* is increasingly diagnosed in diabetic patients with pneumonia/lung infection^1^. It is of note that diabetes is diagnosed in 40–50% of CF patients^11^. Members of the *Pseudomonas* genus are able to adapt to a wide range of environments with over 9% of their large genome encoding regulatory elements, which enables *Pseudomonas* to respond to changes in environmental conditions including nutrient availability^12^,^13^,^14^. *Pseudomonas* has a complex carbohydrate utilisation pathway, with succinate as the preferred carbon source but growth is nicely supported by the availability of glucose. The genes that control the uptake and metabolism of glucose in *P. aeruginosa* are found mostly in an operon which encodes the genes *oprB*, *gltK*, *gtrS* and *glk*, and 3 additional genes encoding a putative ABC transporter^15^. OprB and GltK are involved in glucose transport across the outer and inner membranes, respectively, Glk is the glucokinase that catalyses the reaction of glucose to glucose-6-phosphate for entry into the Entner-Doudoroff pathway and GtrS, together with GltR, forms a two-component regulatory system.

Here we designed an elegant strategy to evaluate the effect of glucose on bacterial lung infection. We investigated the clinical association between hyperglycaemia and airway bacterial colonisation, particularly with *P. aeruginosa*, in the context of critical care. We then generated *P. aeruginosa* mutants in the *oprB*, *gltK*, *gtrS* and *glk* genes and used these mutant strains in *in vitro* and *in vivo* models. The mutants had drastically reduced growth in minimal medium containing glucose as the sole carbon source, whereas they were unaltered when grown in rich medium. In order to explore the effect of elevated glucose with minimal effects on the immune response, we used streptozocin induced diabetes^16^, instead of genetically obese mice (with mutations in leptin signalling), which have a complex phenotype and impaired immune responses^17^. Remarkably, we observed that streptozocin induced hyperglycaemia led to increased bacterial load in the airways when mice were infected with wild type PAO1 but not with the glucose uptake and metabolism mutants. We thus clearly established that increased glucose is a major factor in the increased bacterial infection in diabetes and therefore informed and preventive measures should be taken with these patients.

## Results

### Colonisation of the respiratory tract with organisms among critical care patients is associated with high blood glucose

1,148 admissions to two central London teaching hospital critical care units were identified during the study period (2013–2014); of these 664 had a random blood glucose electronically recorded on admission and were able to be evaluated. Among these 664 patients 241 had a random blood sugar above 11.1 mmol/l and were categorised as high risk for diabetes according to the WHO definitions^18^. After de-duplication 174 patients had samples which grew an organism, 4 of these were yeasts and were discounted leaving 170 patients with bacteria isolated from their sputum in the 7 days succeeding admission. Chi squared analysis demonstrated a statistically significant (p = 0.0002) difference in the likelihood of an organism being isolated between those who had a high blood sugar (of whom 39% had a positive sputum culture) and those who had a low blood sugar (of whom 18% had a positive sputum culture) (Table 1). *P. aeruginosa* was the most frequently isolated organism among critical care patients.

### Deletion of *P. aeruginosa* genes involved in glucose uptake and utilisation attenuates growth when glucose is the sole carbon source

**Figure 1:**
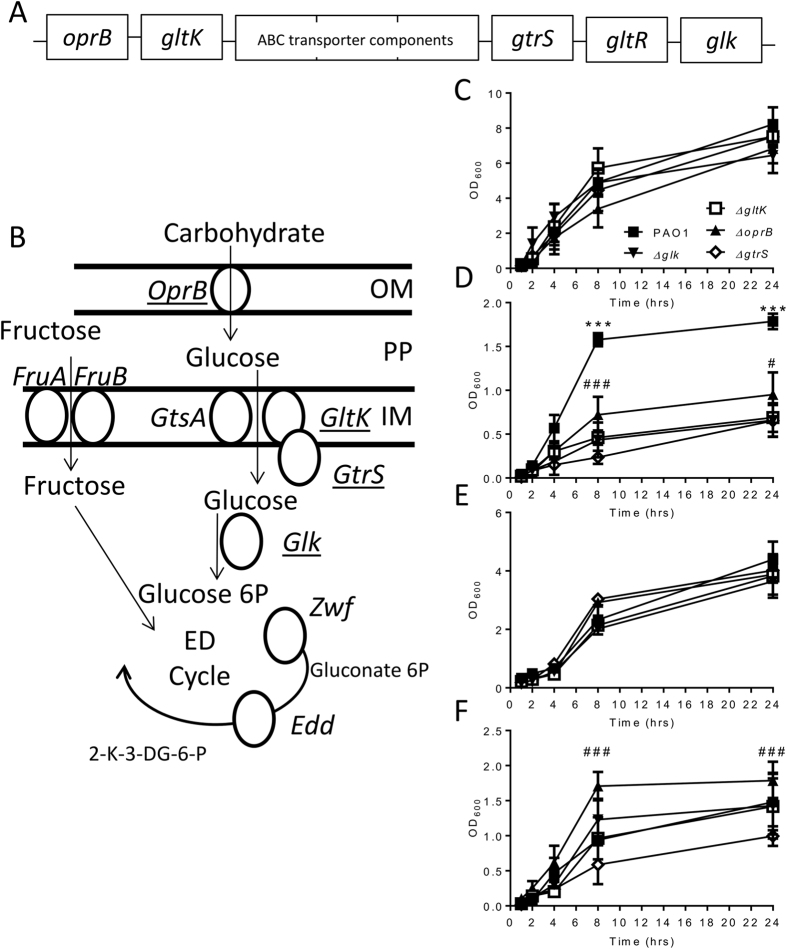
Glucose uptake and utilisation mutants have attenuated growth in minimal medium. *P. aeruginosa* PAO1 glucose uptake operon (**A**). Location of glucose uptake and utilisation genes in *P. aeruginosa* carbon metabolism pathway, genes selected for deletion underlined (**B**). Growth of mutant strains Δ*oprB*, Δ*gltK*, Δ*gtrS* and Δ*glk* was assessed by optical density after inoculation of overnight cultures 1:100 in LB (**C**), M9 with 50 mM glucose (**D**), 50 mM fructose (**E**) or 50 mM succinate (**F**). Points represent the mean +/− SD of n = 3 replicates. *p < 0.01, ***p < 0.001 between PAO1 and other mutants at different glucose concentrations, #p < 0.05, ###p < 0.001 between *ΔoprB* and *ΔgtrS* by two way ANOVA with post test.

Having observed a greater level of bacterial colonisation in patients with high glucose, and a high prevalence of *P. aeruginosa* in these patients, we wished to determine the effect of bacterial glucose uptake and metabolism on colonisation. To test the effect of airway glucose on bacterial growth, we generated *P. aeruginosa* mutants defective in glucose uptake and utilisation (Fig. 1A,B). We engineered clean deletions in either the *oprB*, *gltK*, *gtrS* or *glk* genes using a mutator plasmid based on the pKNG101 suicide vector as described before^19^. The deletion was confirmed using primers designed in the upstream and downstream regions of the targeted gene. We observed no difference in growth between the mutants and parental strain PAO1 in rich medium (LB) (Fig. 1C), indicating that the mutations did not cause any global growth defects. All strains tested entered log phase after 2 hours and growth began to slow by 24 hours after inoculation. Bacteria were unable to grow in minimal medium in the absence of a carbon source (data not depicted). When the different strains were compared using minimal medium supplemented with glucose, there was a significant decrease in growth for the mutants as compared to PAO1 (P < 0.001, Fig. 1D). When minimal media was supplemented with fructose, another monosaccharide, which enters the cell by different proteins bacterial growth was similar between all strains (Fig. 1E). When growth was supplemented with succinate (Fig. 1F), the preferred carbon source for *P. aeruginosa*, the *glk* and *gltK* mutants grew with a similar kinetic to the wild type, but the *gtrS* mutant was significantly attenuated (p < 0.05 at 24 hrs) and interestingly the *oprB* mutant had significantly greater growth. A similar pattern was observed in the glucose supplemented media, with the *oprB* mutant growing slightly better and the *gtrS* mutant growing slightly worse.

**Figure 2:**
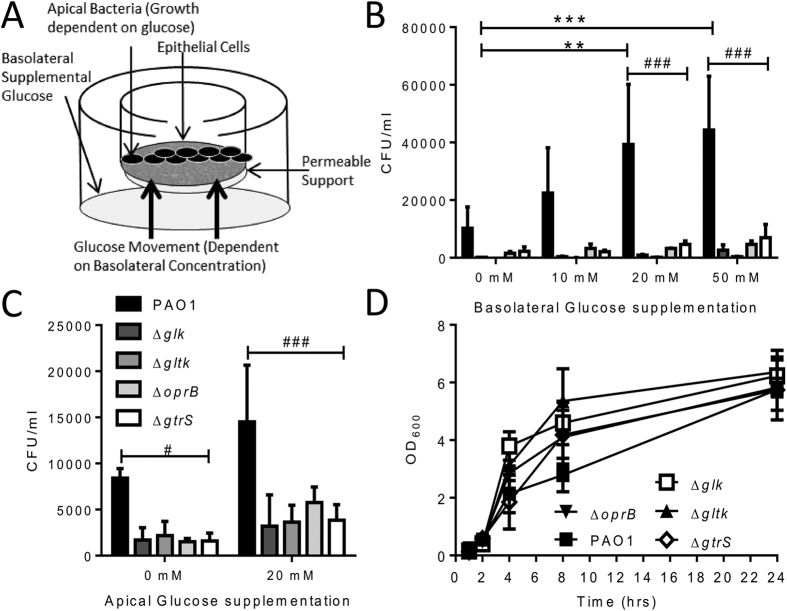
Glucose uptake and utilisation mutants have attenuated growth in airway epithelium co-culture supplemented with glucose. A549 cells were grown on transwell inserts to form monolayers at air liquid interface, transwell resistance measurements were taken to ensure epithelial integrity. The basolateral (**B**) or apical (**C**) concentration of glucose was altered and 10^4^ CFU bacteria added to the apical side and incubated at 37%, 5% CO_2_ for 4 hours. After 4 hours the apical side bacterial numbers were determined by serial dilution. Growth of mutant strains Δ*oprB*, Δ*gltK*, Δ*gtrS* and Δ*glk* was assessed by optical density after inoculation of overnight cultures 1:100 in F12 cell culture media (**D**). Bars represent the mean +/− SD of n = 3 replicates, *p < 0.01, ***p < 0.001 between PAO1 at different glucose concentrations, ###p < 0.001 between wild type PAO1 and the mutants at the same glucose concentration by two way ANOVA with post test.

In previous studies we observed that glucose availability is tightly regulated at epithelial surfaces and that increasing the basolateral glucose can increase apical bacterial growth^8^,^9^. Using a similar model (Fig. 2A), we observed a significantly increased recovery of PAO1 bacteria from the apical surface of airway epithelial monolayers when the basolateral glucose concentration was increased to 50 mM (p < 0.05, Fig. 2B). Growth of the mutant strains was significantly impaired when co-cultured with airway epithelial cells. Increasing either basolateral (Fig. 2B) or apical (Fig. 2C) glucose concentration did not increase apical recovery of the mutants. Glucose appears to be the only carbon source available at the apical surface because the mutant strains grew to the same level as the wild type when grown in the cell culture media used for the studies, without epithelial cells (Fig. 2D). Overall, these data demonstrate that deletion of genes involved in glucose uptake and utilisation reduced the capacity of *P. aeruginosa* to grow when glucose is the sole carbon source.

### Acute hyperglycaemia increases airway bacterial load in wild type but not in glucose uptake and utilisation mutants

**Figure 3:**
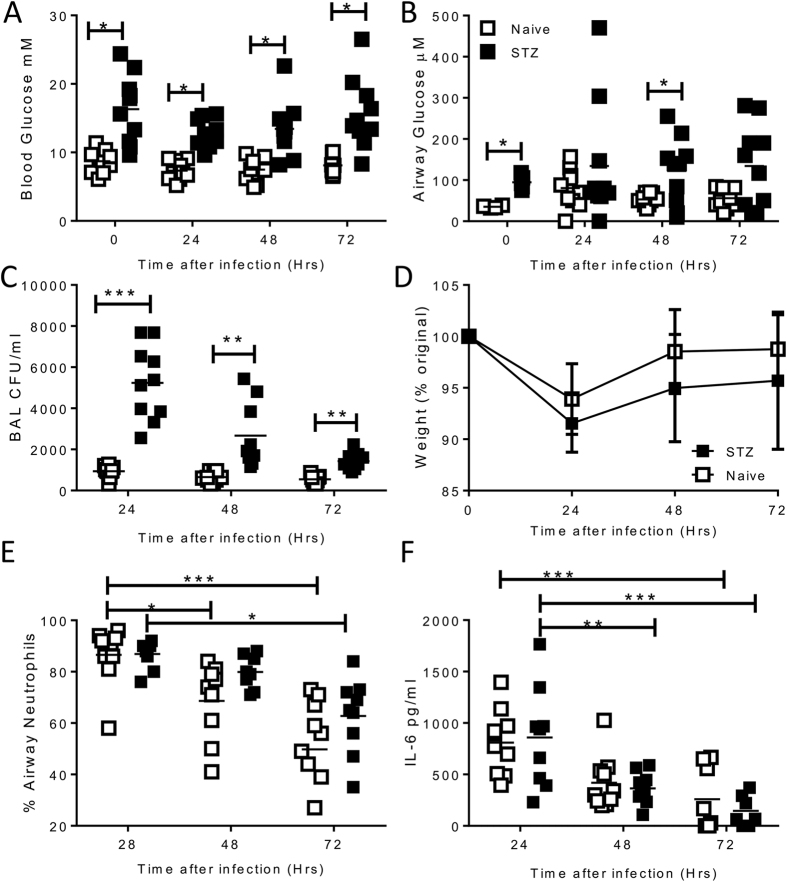
Acute hyperglycaemia leads to increased bacterial load after wild type PAO1 infection. Mice were injected with 2 doses of 10 mg/dose streptozocin intraperitoneally (STZ, closed squares) or untreated (open squares). Glucose was measured in blood (**A**) or BAL (**B**) prior to infection. Log phase growth PAO1 was administered intranasally at a concentration of 10^6^ CFU/mouse. Bacterial burden was measured in BAL (**C**). Weight change was measured (**D**) and percentage airway neutrophils (**E**) and IL-6 were measured in the BAL (**F**). Points represent individual mice (**A–C**,**E**,**F**) or mean +/− SD of n = 5 animals. Lines represent mean values. *p < 0.05, **p < 0.01, ***p < 0.001, calculated using multiple t tests with weighted corrections.

To assess the impact of lung glucose on bacterial lung infection, we induced acute hyperglycaemia in mice prior to infection by treating with streptozocin (STZ). STZ pre-treatment significantly increased blood glucose level concentration (mean STZ 16.3 +/− 1.6 mM, naïve 8.7 +/− 0.5 mM p < 0.001, Fig. 3A). Critically, airway glucose level concentration was significantly increased by STZ pre-treatment (mean STZ 365.7 +/− 26.3 μM, naïve 105.7 +/− 10.6 μM p < 0.001, Fig. 3B). Mice were infected with 10^6^ CFU of log phase *P. aeruginosa* in a 100 μl volume intranasally (i.n.), pre-treatment with STZ significantly increased the amount of recovered bacteria from the airways (measured in bronchoalveolar lavage: BAL) at 24, 48 and 72 hours after infection, delaying bacterial clearance (p < 0.01, Fig. 3C). The glucose levels in the blood were consistently higher in the STZ treated mice than the naïve mice throughout the experiment. Markers of an inflammatory immune response were similar in both groups, independent of hyperglycaemia. Both groups of animals displayed an acute, transitory weight loss (Fig. 3D) with cell recruitment to the airways consisting mostly of neutrophils (Fig. 3E), the proportion of which declined as the infection was cleared in the naïve group, but more slowly in the STZ group, potentially reflecting the delayed bacterial clearance. Infection significantly increased levels of the pro-inflammatory cytokine IL-6 at 24 hours after infection in the airways (Fig. 3F), which declined as the infection was cleared.

**Figure 4:**
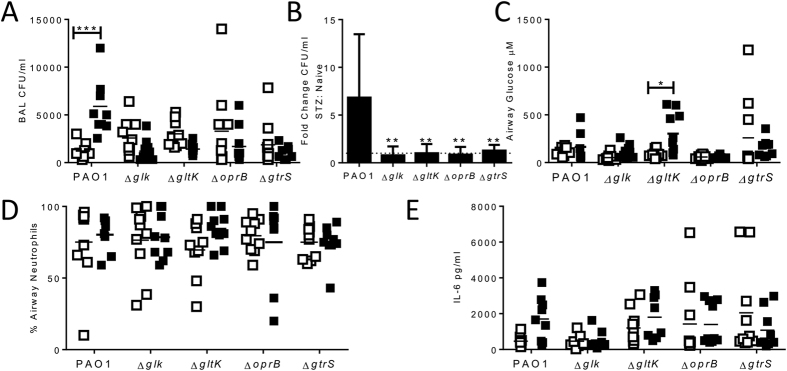
Hyperglycaemia increases acute bacterial load in mice infected with WT but not knockout strains. Mice were injected with 2 doses of 10 mg/dose Streptozocin (STZ) intraperitoneally, and mice were infected intranasally with 10^6^ CFU/mouse of either WT PAO1 or mutant strains and sacrificed after 24 hours. BAL CFU (**A**), fold change in BAL CFU between STZ and control animals (**B**), BAL neutrophilia (**C**) and IL-6 (**D**) were assessed at 24 hours after infection. Points represent individual mice, lines represent mean values, bars represent mean +/− SEM of n = 5 animals. *p < 0.05, calculated using multiple t tests with weighted corrections.

We then compared the growth of the glucose mutants with the parental strain PAO1 in STZ treated or control naïve mice. This experiment readily showed that there were far more PAO1 bacteria recovered from the airways in STZ treated as compared to naive mice (p < 0.05, Fig. 4A). However, STZ induced hyperglycaemia did not increase the recovery of the glucose mutants, the fold change between naïve and STZ treated was significantly greater for PAO1 infected animals(Fig. 4B). In the naïve (STZ untreated) mice, there was no difference in the bacterial load following infection with either the parental PAO1 or the mutant bacteria. The airway glucose was significantly greater during GltK infection of STZ treated mice than naïve animals. Beyond the striking differences in airway bacterial load in STZ treated animals, there was no difference when using different bacteria in terms of the inflammatory responses, with similar neutrophil recruitment (Fig. 4D) and inflammatory cytokine responses (Fig. 4E). Overall, these data show that streptozocin-induced hyperglycaemia in mice led to a higher bacterial burden on infection which is linked directly with the ability of the bacteria to metabolise glucose.

### Treatment with metformin significantly reduces airway bacterial load in hyperglycaemic mice

**Figure 5:**
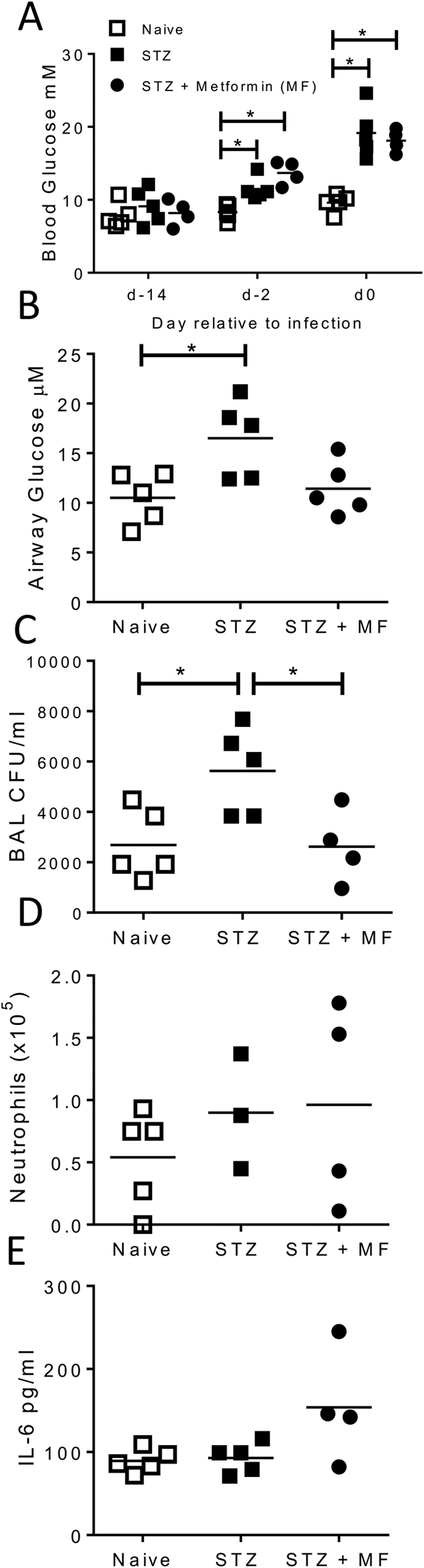
Metformin reduces airway bacterial load after wild type PAO1 infection in hyperglycaemic mice. Mice were injected with 2 doses of 10 mg/dose Streptozocin (STZ) intraperitoneally. On d-2, d-1 and d0 of infection, one group of mice received a 200 μl dose of 4 mg/ml i.p. metformin (MF). Blood glucose was measured before and after drug treatment (**A**). BAL glucose was measured after drug treatment (**B**). Mice were infected intranasally with 10^6^ CFU/mouse of WT PAO1 and sacrificed after 24 hours. BAL CFU (**C**), neutrophilia (**D**) and IL-6 (**E**) were assessed at 24 hours after infection. Points represent individual mice. Lines represent mean values. *p < 0.05, calculated using multiple t tests with weighted corrections.

Controlling airway glucose may provide a novel approach to control bacterial lung infection in diabetes. To test this we used the anti-diabetic drug metformin. After induction of hyperglycaemia using streptozocin, mice were treated with metformin prior to infection. Streptozocin treatment significantly increased blood (Fig. 5A) and airway (Fig. 5B) glucose prior to infection. Metformin treatment did not change blood glucose levels but reduced BAL glucose levels to the same as naïve animals. Strikingly, pre-treatment with metformin led to a significant reduction in the airway bacterial load (Fig. 5C, p < 0.05). There was no significant difference in the number of neutrophils (Fig. 5D) or IL-6 (Fig. 5E) regardless of treatment regime.

## Discussion

In this study we demonstrated a clinical association between high blood glucose and respiratory tract bacterial colonisation. We also show that hyperglycaemia induced increases in airway surface liquid glucose concentration were associated with increased airway bacterial load, using *P. aeruginosa* with targeted gene deletions in the glucose metabolism pathway. The use of the *P. aeruginosa* mutants is key and instrumental to point out the direct relationship between bacterial load and the ability to use glucose or not. The genes we chose to delete are involved in various stages of the uptake and metabolism of glucose, which ensures specificity and avoids pleotropic effects. The *in vitro* growth of these mutants was shown to be identical to the parental strain in LB or minimal medium supplemented with fructose, whereas growth was drastically affected when glucose was the only carbon source. Interestingly, the *gtrS* mutant was further attenuated in either succinate or glucose supplemented media, which may reflect the gtrS protein’s role as a regulator. The recovered CFU of the PAO1Δ*oprB* strain was higher than PAO1 in succinate supplemented minimal media, which may be caused by the relaxation of catabolite repression in the mutant strain^20^. The genes deleted in this study were highly targeted as compared to previous studies which used *edd* gene mutants of *P. aeruginosa*^6^, which are affected in the metabolism of several different carbon sources, not only glucose^21^ and were unable to grow in either control or hyperglycaemic animals.

Using *in vitro* airway epithelia-bacteria co-cultures, we observed a significant reduction in the growth of all glucose mutants. Compared to the parental strain, bacterial recovery after infection with the mutants was lower even when glucose was increased. There was no difference in bacterial growth observed in the F12 media alone, which in addition to glucose contains pyruvate and amino acids. Taken together, this suggests that the main utilisable carbon source for *P. aeruginosa* at the airway epithelial surface in the co-culture system *in vitro* is glucose. However, in normal mouse lungs the glucose mutants were not attenuated compared to the parental strain, which suggests that unlike the *in vitro* system, there are other carbon sources available in the lungs. Interestingly airway glucose remained significantly higher 24 hours after infection in STZ treated Δ*gltK* infected animals than naïve animals infected with the same bacteria, possibly reflecting the role of GltK as glucose uptake pore. Understanding what other carbon sources are available to bacteria in the lungs may open up alternative intervention strategies.

Controlling airway glucose concentration is one example of a number of non-immune mechanisms that have evolved to prevent bacterial infection. Glucose is not the only nutrient that is restricted in the airways, levels of amino acids and other carbon sources are also limited^22^. Interactions and competition between microbes for this limited resource can lead to altered virulence of the bacteria^23^. Glucose is also important in host immune function and bacterial exposure bacteria can increase cellular glucose uptake^9^, the competition between host cells and bacteria for airway nutrients is of interest and needs further research, especially the effect of metformin on the host immune cells. In addition to directly affecting bacterial growth and survival, airway surface liquid chemistry has an effect on the secretion and action of antimicrobial molecules. Airway surface pH has been demonstrated to be critical for the function of antimicrobial activity in the lungs^24^. Glucose can inhibit the secretion of anti-microbial peptides in the airways induced by bitter taste receptors^25^ and glucose inhibits the function of surfactant protein D^26^.

There were a number of experimental limitations that need to be considered. One of the limitations of the study is that we were not able to separate the effect of elevated glucose on the host response from the effect of elevated glucose on the bacteria. It has previously been asserted that increased bacterial infection in diabetes is driven by underlying immunodeficiencies^27^ and it is likely that elevated glucose levels alters the immune response. Whilst we only performed a limited analysis of the immune response, we saw no differences in the immune parameters measured, airway neutrophilia and IL-6, between the hyperglycaemic and control animals. But we did, however, observe an association between elevated airway glucose and bacterial load, which was only seen in glucose metabolism competent bacteria. A second limitation is that streptozocin induced hyperglycaemia in mice is not the same as diabetes in patients: however, similar increases in bacterial loads have been observed in two other models of hyperglycaemia, leptin (*ob/ob*)^6^ and leptin receptor (*db/db*)^8^ deficient mice. Finally, the mutants we generated were only specific for glucose and therefore, it may be that other carbon sources are elevated in the airways in hyperglycaemia enabling bacterial growth, which is of interest and would need to be tested in other studies. Even taking these limitations into account we still believe that our work strongly supports the hypothesis that elevated airway glucose drives increased bacterial growth.

The manipulation of airway surface liquid chemistry may offer alternative avenues to control lung bacterial infection than conventional antibiotics, which are becoming less effective for many of the main causes of bacterial respiratory infection. Treatment with metformin reduced airway glucose levels in hyperglycaemic animals and significantly reduced bacterial load in the airways. We have recently observed a similar effect after *S. aureus* infection in obese mice treated with metformin^8^, demonstrating the breadth of this approach as it can reduce bacterial load after infection with either gram positive or negative bacteria. Metformin usage is safe in patients with COPD^28^ and an ongoing clinical trial is exploring whether metformin can reduce COPD exacerbations (NCT01247870). These approaches have the advantage of targeting host mechanisms, which may be harder for the pathogen to escape. These new findings thus indicate that hyperglycaemia has the potential to drive respiratory infection in the absence of other hallmarks of diabetic disease with implications for human health. Our work thus opens up novel strategic targets for the control of bacterial lung infection in the most at risk patients.

## Methods

### Critical Care admissions database

An established anonymised research database was interrogated to elicit demographic information, blood glucose levels (highest recorded on day of critical care admission) and culture positive microbiology results during the first 7 days of the critical care admission. All admissions to two central London teaching hospital critical care units (belonging to a hospital network with overarching governance structures, policies and who share a centralised laboratory which observes national standard operating procedures) were included for the fiscal year 2013–2014 (in the UK fiscal years run April to March). Where multiple isolates were grown, only the first was selected. Extraction, pseudoanonymisation, and analysis of the clinical epidemiological data in this study was conducted after review by, and with the approval of, the Imperial College London Joint Research Compliance Office (ref. 13 HH0747).

### Gene deletion

The laboratory strain PAO1^29^ was used throughout this study. Deletion of glucose uptake and utilisation genes (*oprB*, *gltK*, *gtrS* and *glk*) in the PAO1 background was generated as previously described^19^. Briefly, PCR amplification of DNA flanking the gene of interest was used to generate a mutator fragment which was then cloned into the suicide vector PKNG101 carrying the gene conferring Sm^R^ and the sucrose selection gene *sacB*. Through a series of recombination events the gene of interest is deleted and the insert removed upon selection on sucrose-containing plates. Successful gene deletion was confirmed by PCR.

### Bacterial growth characterisation

Bacteria were plated out on Lysogeny Broth (LB) agar (Sigma) to single colonies prior to overnight growth in 5 ml liquid LB. 1:100 dilution of overnight culture was inoculated into 5 ml LB and growth assessed by optical density (OD_600_). Bacterial growth was assessed in LB and M9 minimal salts media supplemented with 50 mM succinate or glucose where indicated.

### Polarised airway epithelia-bacteria co-cultures

A549 cells were cultured and grown in F-12 media supplemented with fetal calf serum and L-glutamine on transwell inserts. Prior to infection, transwell resistance (R_T_) measurements were taken to ensure epithelial integrity using an Ohmmeter (Millicell ERS-2 Ohmmeter) and rejected if < 100Ω. *P. aeruginosa* was prepared by inoculating fresh LB with overnight culture and grown to a log phase OD_600_ of 0.4 log phase (approximately 10^7^ CFU/ml determined by confirmed by plating out serial dilutions). Cultures were diluted to 10^5^ CFU/ml in PBS and 100 μl applied to the apical side. Co-cultures were incubated at 37 °C, 5% CO_2_ for 4 hours. After 4 hours the epithelial cells and bacteria were homogenised and serially diluted to determine CFUs from the apical side.

### *In vivo* models

6–8 week old female C57BL/6 mice were obtained from Harlan UK Ltd (Logosenn, Truro, UK) and kept in specific-pathogen-free conditions in accordance with the United Kingdom’s Home Office guidelines and approved by the Animal Welfare and Ethical Review Board (AWERB) at Imperial College London. Mice were infected with 10^6^ CFU of log phase *P. aeruginosa* in a 100 μl volume intranasally (i.n.). Where performed, hyperglycaemia was induced using the drug streptozocin (STZ) which was administered by intraperitoneal (i.p.) injection at a concentration of 10 mg/mouse in 0.5 ml on day −14 and day −7 prior to infection. Glucose was monitored by taking blood from the tails of mice and using a OneTouch UltraEasy blood glucose monitor and OneTouch UltraEasy Test Strips (LifeScan, UK) and urine glucose was monitored using Diastix reagent sticks (Bayer Diagnostic Fisher). BAL glucose was measured post infection by High Performance Liquid Chromatography (HPLC) as previously described^30^, or amplex red glucose/glucose oxidase assay kit (Invitrogen). When used, mice were pre-treated with 200 μl 4 mg/ml of metformin (Sigma) i.p. on days −2, −1 and 0 prior to infection.

### Tissue and cell recovery and isolation

Mice were culled using i.p. pentobarbitone. Bronchoalveolar lavage (BAL) was obtained by inflating the lungs via an intratracheal cannula with PBS. Lungs were removed and homogenised by passage through 100 μm cell strainers. Bacterial loads were determined in untreated BAL and lung homogenate by serial dilution as described^31^ on Pseudomonas isolation agar (Sigma, UK) using a multichannel pipette. After plating for bacterial load, lung samples and BAL were treated with red blood cell lysis buffer before centrifugation at 200 × *g* for 5 minutes. Cells were resuspended in RPMI medium with 10% fetal calf serum and viable cell numbers determined by trypan blue exclusion. For differential cell counts, 100 μl of cells from BAL and the lung homogenate were centrifuged onto glass slides. Samples were air dried, and fixed in methanol, before staining of with haematoxylin and eosin. Cytokines were assessed by ELISA (R&D systems) following manufacturer’s instructions.

### Statistical analysis

Clinical data were statistically analysed using Chi squared or Fisher’s exact test as appropriate in STATA^®^ IC 13 (TX). For the *in vitro* and *in vivo* models comparisons of two groups were performed using Student’s t tests. Comparisons of multiple groups were performed using one- or two-way ANOVA with appropriate post-tests. All statistical tests were performed using GraphPad Prism version 6.01 for Windows (GraphPad Software, San Diego California USA).

## Additional Information

**How to cite this article**: Gill, S. K. *et al.* Increased airway glucose increases airway bacterial load in hyperglycaemia. *Sci. Rep.* **6**, 27636; doi: 10.1038/srep27636 (2016).

## Figures and Tables

**Table 1:** Association between blood glucose at admission to critical care and bacterial colonisation of the lung in the first 7 days of admission.

	Highest glucose on day of ICU admission ≥ 11.1 mmol/l	Highest glucose on day of ICU admission <11.1 mmol/l
Number	241	423
Mean Age	62.6 years	69.6 years
Proportion female	41%	47%
Admission category		
Medicine	62%	57%
Surgery	38%	43%
Culture positive sputum sample in the first 7 days of critical care admission	94 (39%) *p* = 0.0002	76 (18%)
Organism isolated		
*Pseudomonas* spp.	30 (32%)	20 (26%)
*Staphylococcus aureus*	13	12
*Klebsiella* spp.	12	11
*Escherichia coli*	12	9
*Enterobacter* spp.	11	6
*Stenotrophomonas maltophilia*	7	3
*Serratia marcescens*	3	6
Streptococci	0	6
*Citrobacter* spp.	3	1
*Proteus* spp.	2	1
*Acinetobacter* spp.	1	0
*Morganella morganii*	0	1

## References

[b1] Baker E. *et al.* Hyperglycemia and cystic fibrosis alter respiratory fluid glucose concentrations estimated by breath condensate analysis. *J. Appl physiol* (*Bethesda, Md.: 1985*) 102, 1969–1975, 10.1152/japplphysiol.01425.2006 (2007). 17303703

[b2] Philips B. *et al.* Glucose in bronchial aspirates increases the risk of respiratory MRSA in intubated patients. Thorax 60, 761–764, 10.1136/thx.2004.035766 (2005). 16135681 PMC1747508

[b3] Baker E. H. *et al.* Hyperglycaemia and pulmonary infection. Proc.Nutr.Soc. 65, 227–235, http://dx.doi.org/10.1079/PNS2006499 (2006). 16923307 10.1079/pns2006499

[b4] Rueda A. M. *et al.* Hyperglycemia in diabetics and non-diabetics: Effect on the risk for and severity of pneumococcal pneumonia. J Infect 60, 99–105, http://dx.doi.org/10.1016/j.jinf.2009.12.003 (2010). 20005251 10.1016/j.jinf.2009.12.003

[b5] Philips B., Meguer J.-X., Redman J. & Baker E. Factors determining the appearance of glucose in upper and lower respiratory tract secretions. ICM 29, 2204–2210, 10.1007/s00134-003-1961-2 (2003). 14647890

[b6] Pezzulo A. *et al.* Glucose depletion in the airway surface liquid is essential for sterility of the airways. PloS one 6, e16166, 10.1371/journal.pone.0016166 (2011). 21311590 PMC3029092

[b7] Kalsi K. K. *et al.* Apical and basolateral localisation of GLUT2 transporters in human lung epithelial cells. Pflugers Arch 456, 991–1003, 10.1007/s00424-008-0459-8. (2008). 18239936 PMC2480509

[b8] Garnett J. *et al.* Metformin reduces airway glucose permeability and hyperglycaemia-induced Staphylococcus aureus load independently of effects on blood glucose. Thorax. 68, 835–45, 10.1136/thoraxjnl-2012-203178 (2013). 23709760 PMC3756442

[b9] Garnett J. P. *et al.* Elevated paracellular glucose flux across cystic fibrosis airway epithelial monolayers is an important factor for Pseudomonas aeruginosa growth. PloS one 8, e76283, 10.1371/journal.pone.0076283 (2013). 24124542 PMC3790714

[b10] Williams B., Dehnbostel J. & Blackwell T. Pseudomonas aeruginosa: host defence in lung diseases. Respirology 15, 1037–1056, 10.1111/j.1440-1843.2010.01819.x (2010). 20723140

[b11] van den Berg J. M., Kouwenberg J. M. & Heijerman H. G. Demographics of glucose metabolism in cystic fibrosis. J Cyst Fibros 8, 276–279, 10.1016/j.jcf.2009.04.010 (2009). 19467621

[b12] Silby M., Winstanley C., Godfrey S., Levy S. & Jackson R. Pseudomonas genomes: diverse and adaptable. FEMS microbiol rev 35, 652–680, 10.1111/j.1574-6976.2011.00269.x (2011). 21361996

[b13] Mathee K. *et al.* Dynamics of Pseudomonas aeruginosa genome evolution. PNAS, 105, 3100–3105, 10.1073/pnas.0711982105 (2008). 18287045 PMC2268591

[b14] Nguyen D. & Singh P. Evolving stealth: genetic adaptation of Pseudomonas aeruginosa during cystic fibrosis infections. PNAS, 103, 8305–8306, 10.1073/pnas.0602526103 (2006). 16717189 PMC1482488

[b15] Winsor G. L. *et al.* Pseudomonas Genome Database: improved comparative analysis and population genomics capability for Pseudomonas genomes. NAR, 39, D596–600, 10.1093/nar/gkq869 (2011). 20929876 PMC3013766

[b16] Deeds M. C. *et al.* Single dose streptozotocin-induced diabetes: considerations for study design in islet transplantation models. Laboratory animals 45, 131–140, 10.1258/la.2010.010090 (2011). 21478271 PMC3917305

[b17] Mancuso P. *et al.* Leptin-Deficient Mice Exhibit Impaired Host Defense in Gram-Negative Pneumonia. J Immunol 168, 4018–4024, 10.4049/jimmunol.168.8.4018 (2002). 11937559

[b18] WHO & IDF. Definition and diagnosis of diabetes mellitus and intermediate hyperglycaemia. Report of a WHO/IDF consultation. http://www.who.int/diabetes/publications/diagnosis_diabetes2006/en/ (2006).

[b19] Ruer S., Stender S., Filloux A. & de Bentzmann S. Assembly of fimbrial structures in Pseudomonas aeruginosa: functionality and specificity of chaperone-usher machineries. J bacteriol 189, 3547–3555, 10.1128/JB.00093-07 (2007). 17293418 PMC1855894

[b20] Valentini M. & Lapouge K. Catabolite repression in Pseudomonas aeruginosa PAO1 regulates the uptake of C4 -dicarboxylates depending on succinate concentration. Envi microbiol 15, 1707–1716, 10.1111/1462-2920.12056 (2013). 23253107

[b21] Kim H. J. *et al.* Mutation in the edd gene encoding the 6-phosphogluconate dehydratase of Pseudomonas chlororaphis O6 impairs root colonization and is correlated with reduced induction of systemic resistance. Lett Appl Microbiol 44, 56–61, 10.1111/j.1472-765X.2006.02029.x (2007). 17209815

[b22] Krismer B. *et al.* Nutrient limitation governs Staphylococcus aureus metabolism and niche adaptation in the human nose. PLoS pathogens 10, e1003862, 10.1371/journal.ppat.1003862 (2014). 24453967 PMC3894218

[b23] Vandanmagsar B. *et al.* The NLRP3 inflammasome instigates obesity-induced inflammation and insulin resistance. Nature medicine 17, 179–188, 10.1038/nm.2279 (2011). PMC3076025 21217695

[b24] Pezzulo A. *et al.* Reduced airway surface pH impairs bacterial killing in the porcine cystic fibrosis lung. Nature 487, 109–113, 10.1038/nature11130 (2012). 22763554 PMC3390761

[b25] Lee R. J. *et al.* Bitter and sweet taste receptors regulate human upper respiratory innate immunity. JCI 124, 1393–1405, 10.1172/JCI72094 (2014). 24531552 PMC3934184

[b26] Reading P. C., Allison J., Crouch E. C. & Anders E. M. Increased Susceptibility of Diabetic Mice to Influenza Virus Infection: Compromise of Collectin-Mediated Host Defense of the Lung by Glucose? J Virol 72, 6884–6887, doi: http://jvi.asm.org/content/72/8/6884 (1998). 9658139 10.1128/jvi.72.8.6884-6887.1998 PMC109899

[b27] Hodgson K. *et al.* Immunological mechanisms contributing to the double burden of diabetes and intracellular bacterial infections. Immunol 144, 171–185, 10.1111/imm.12394 (2014). PMC4298412 25262977

[b28] Hitchings A. W., Archer J. R., Srivastava S. A. & Baker E. H. Safety of Metformin in Patients with Chronic Obstructive Pulmonary Disease and Type 2 Diabetes Mellitus. COPD 2, 126–131, 10.3109/15412555.2014.898052 (2014). 25938184

[b29] Holloway B. W. Genetic recombination in Pseudomonas aeruginosa. J Gen Microbiol 13, 572–581, 10.1099/00221287-13-3-572 (1955). 13278508

[b30] Kalsi K. *et al.* Glucose homeostasis across human airway epithelial cell monolayers: role of diffusion, transport and metabolism. Pflugers Arch 457, 1061–1070, 10.1007/s00424-008-0576-4 (2009). 18781323

[b31] Siggins M. K. *et al.* PHiD-CV induces anti-Protein D antibodies but does not augment pulmonary clearance of nontypeable *Haemophilus influenzae* in mice. Vaccine 33, 4954–4961, 10.1016/j.vaccine.2015.07.034 (2015). 26212006

